# Characterization and evaluation of an integrated quality monitoring system for online quality assurance of external beam radiation therapy

**DOI:** 10.1002/acm2.12014

**Published:** 2016-11-29

**Authors:** David Hoffman, Eunah Chung, Clayton Hess, Robin Stern, Stanley Benedict

**Affiliations:** ^1^ Department of Radiation Medicine and Applied Sciences University of California San Diego CA USA; ^2^ Department of Radiation Oncology Samsung Medical Center Seoul South Korea; ^3^ Pediatric Radiation Oncology Harvard Medical School Boston MA USA; ^4^ Department of Radiation Oncology University of California Davis, Sacramento CA USA

**Keywords:** external beam radiation therapy, ion chamber, quality assurance

## Abstract

**Purpose:**

The aim of this work was to comprehensively evaluate a new large field ion chamber transmission detector, Integral Quality Monitor (IQM), for online external photon beam verification and quality assurance. The device is designed to be mounted on the linac accessory tray to measure and verify photon energy, field shape, gantry position, and fluence before and during patient treatment.

**Methods:**

Our institution evaluated the newly developed ion chamber's effect on photon beam fluence, response to dose, detection of photon fluence modification, and the accuracy of the integrated barometer, thermometer, and inclinometer. The detection of photon fluence modifications was performed by measuring 6 MV with fields of 10 cm × 10 cm and 1 cm × 1 cm “correct” beam, and then altering the beam modifiers to simulate minor and major delivery deviations. The type and magnitude of the deviations selected for evaluation were based on the specifications for photon output and MLC position reported in AAPM Task Group Report 142. Additionally, the change in ion chamber signal caused by a simulated IMRT delivery error is evaluated.

**Results:**

The device attenuated 6 MV, 10 MV, and 15 MV photon beams by 5.43 ± 0.02%, 4.60 ± 0.02%, and 4.21 ± 0.03%, respectively. Photon beam profiles were altered with the IQM by < 1.5% in the nonpenumbra regions of the beams. The photon beam profile for a 1 cm × 1 cm^2^ fields were unchanged by the presence of the device. The large area ion chamber measurements were reproducible on the same day with a 0.14% standard deviation and stable over 4 weeks with a 0.47% SD. The ion chamber's dose–response was linear (R^2^ = 0.99999). The integrated thermometer agreed to a calibrated thermometer to within 1.0 ± 0.7°C. The integrated barometer agreed to a mercury barometer to within 2.3 ± 0.4 mmHg. The integrated inclinometer gantry angle measurement agreed with the spirit level at 0 and 180 degrees within 0.03 ± 0.01 degrees and 0.27 ± 0.03 at 90 and 270 degrees. For the collimator angle measurement, the IQM inclinometer agreed with a plum‐bob within 0.3 ± 0.2 degrees. The simulated IMRT error increased the ion chamber signal by a factor of 11–238 times the baseline measurement for each segment.

**Conclusions:**

The device signal was dependent on variations in MU delivered, field position, single MLC leaf position, and nominal photon energy for both the 1 cm × 1 cm and 10 cm × 10 cm fields. This detector has demonstrated utility repeated photon beam measurement, including in IMRT and small field applications.

## 1 Introduction

Radiation therapy has increased in complexity in recent years due to substantial advances in novel treatment planning and delivery techniques. Intensity modulated radiation therapy (IMRT),^1^ tomotherapy,^2^ image‐guided radiation therapy (IGRT),^3^, ^4^ volumetric‐modulated arc therapy (VMAT),^5^, ^6^ and small field treatments utilized in SRT and SBRT are examples of radiation delivery practice with more intricate work flow than “conventional” radiation therapy. Due to this increased complexity, new quality assessment (QA) strategies have been developed, including patient‐specific dose verification.^7^, ^8^, ^9^, ^10^ This patient‐specific QA measurement is only performed once for a treatment course that can include as many as 44 sessions. During the course of treatment, errors may be introduced by changes in software, hardware, or human procedure. One strategy to address these potential treatment errors is online monitoring of every radiation therapy session. This goal can be accomplished by placing a transmission detector on the head of a linac and making dosimetric measurements of the radiation beams as they are being delivered to the patient. This online monitoring has potential to detect many potential treatment errors.^11^

The value and importance of performing a measurement of the radiation delivered for each fraction of a course of external beam radiation therapy has been previously discussed by Mijnheer et al.^12^ In fact, a dosimetry measurement is required by the national recommendations of Sweden and France, and is recommended by Royal College of Radiologists of the United Kingdom.^12^ In this context, transmission detector systems will likely become a more prevalent quality assurance measure in the future.

Online photon beam dose verification with a transmission detector system has been previously demonstrated. Paliwal et al.^13^ used a large area transparent transmission chamber mounted on the shielding tray that detected deviations from the initial treatment in photon beam fluence in subsequent sessions. Another strategy is the use of a flat, multi‐wire transmission‐type ionization chamber, attached to the accessory holder of a linac.^14^ One such system is known as the DAVID system (PTW‐Freiburg, Freiburg, Germany) and has evaluated for the online detection of MLC discrepancies in IMRT deliveries.^15^ COMPASS^®^ (IBA Dosimetry, Schwarzenbruck, Germany) is a transmission detector consisting of 1600 plane‐parallel ionization chambers.^16^ It has been used for the online measurement of IMRT treatments and validated by Monte Carlo simulation.^17^ Islam et al. developed an area integrating energy fluence monitoring sensor (AIMS) capable of detecting errors in MLC leaf calibration or malfunctions in the positioning of an individual leaf^18^ as well as verification of adapted treatment fields.^19^ Another transmission detector, named the “magic plate,” has been developed using a 2D array of silicon diodes.^20^, ^21^ The VANILLA system uses monolithic active pixel sensors to measure ionizing radiation beam profiles.^22^ Another monitor has been developed that utilized optical attenuation‐based detectors to measure light produced in long scintillating fibers by the photon fluence at the linac head.^23^

This work characterized the *Integral Quality Monitor* (*IQM*), developed by iRT Systems GmbH (Koblenz, Germany). The design of this commercially available device is based on the research prototype developed by Islam et al.^18^ The aim of this work was to evaluate the IQM's effect on photon beam fluence, response to dose, detection of photon fluence modification, and the accuracy of the integrated barometer, thermometer, and inclinometer. Additionally, this research evaluates the dependence of the ion chamber's signal on MLC and photon beam characteristics selected based on the quality assurance recommendations of AAPM Task Group 142.^24^ This publication represents original research, different from the previously published work of Islam et al. in that: 1) The earlier publication was for prototype device with a ion chamber and electronics design that was never commercially available, 2) This work utilized a new commercially available design that operates as a bluetooth wireless device, 3) the integrated inclinometer, barometer, and thermometer is evaluated, 4) the effect on photon beam percent depth dose (PDD) and profile is evaluated for energies beyond 6 MV, including 10 and 15 MV, 5) the scenario of the device losing power mid‐treatment is evaluated, 6) the effect on photon beam profile for a IMRT sized field (1 × 1 cm^2^) is characterized, 7) a dose‐rate dependence of the ion chamber response is evaluated and addressed, 8) the change in ion chamber signal caused by a simulated IMRT delivery error is evaluated.

## 2 Material and method

**Figure 1:**
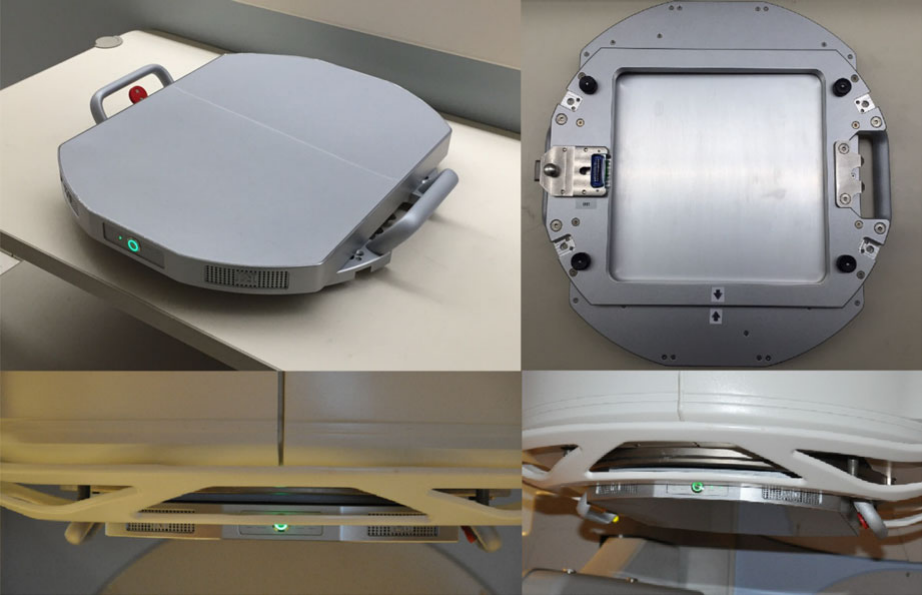
The Integral Quality Monitor (IQM) is a large area ion chamber (top left) with a gradient of the ion chamber thickness in the axis of MLC motion. It attaches to the accessory tray holder, similar to an electron cone (top right). The device has a low profile from the linac head (bottom left and right) and connects wirelessly to a transceiver and the controlling computer.

The IQM is a commercially available quality monitoring system composed of a large area (26 cm × 26 cm) position‐sensitive ion chamber, barometer, thermometer, and inclinometer. The device attaches to the accessory tray holder of a linear accelerator, as shown in Fig. 1, and connects wirelessly to a transceiver and the controlling computer. Photon treatment beams pass through the active volume of the ion chamber during patient treatment and for QA measurements. The ion chamber collects charge produced by the photon beam and reports a total for each beam, control point, or segment, depending on treatment modality. This total is corrected for temperature and pressure variations and reported in arbitrary units (counts) that serve as a checksum for each photon beam treatment. A gradient in the ion chamber active volume thickness in the MLC motion axis makes the magnitude of the signal dependent on the beam position in the gradient direction. Additionally, the device monitors gantry and collimator angles by the inclination of the device as measured with the integrated inclinometer. The IQM data acquisition software system is interfaced with the linear accelerator to access patient‐specific treatment parameter information, including field number, field name, and delivery type.

The creation of a checksum is an important aspect of the IQM's function. The digitized current produced in the IQM's ion chamber is recorded for every beam, control point, or segment during radiation delivery. At the end of a treatment session, each measurement, as well as the total signal, or checksum, can be compared to previous measurements. This baseline measurement could be performed during a patient‐specific quality assurance measurement, before delivery of the first fraction. This allows for outlying deliveries to be quickly detected, possible even during treatment delivery.

### 2.A Integrated quality monitoring system evaluation

To validate the large area detector's ability to make useful EBRT quality assurance measurements, the sensitive area of the detector should be sufficiently sized to intersect all linear accelerator beams. The linear accelerator used for this work was an *Elekta Synergy* (Stockholm, Sweden) with an *Agility* MLC to produce photon beams with nominal energies of 6 MV, 10 MV, and 15 MV. Varian (Palo Alto, CA, USA) linac compatible devices are also commercially available. Adequate coverage of the full range of MLC and jaw motion was evaluated by serial measurement of IQM signal initially for a 24 cm × 24 cm field (100 MU) and then for incrementally larger field sizes, with horizontal and vertical axis length being increased separately. Increased signal with increasing field size was interpreted as adequate coverage of the jaw aperture by the ion chamber active area. No increase was interpreted as inadequate coverage.

To correct for temperature and pressure effects of the ion chamber measurement, the *IQM* has an integrated thermometer and barometer. The accuracy of these components was evaluated by comparison with an ISO 17025 calibrated, hand‐held thermometer, and a mercury barometer (Princo, Southampton, NY, USA). Both the IQM barometer and the mercury barometer read out mmHg, so this unit is reported. The device also measures gantry angle and collimator angle with an inclinometer. The inclinometer reading was compared with gantry angle as measured with a spirit level at 0, 90, 180, and 270 degrees, and a digital level calibrated to the spirit level at 15 degree increments between those angles. The collimator angle measurement was compared with a plum‐bob at 0, 90, 180, and 270 degrees while the gantry was at 0 and 90 degrees.

To evaluate the large area detector's signal linearity, dose‐rate dependence, reproducibility, and stability, a 6 MV photon 10 cm × 10 cm field was delivered through the center of the active area while modulating total MU and dose rate. A measurement of 100 MU was repeated 10 times to evaluate reproducibility. The same measurement was repeated 9 times over 4 weeks to evaluate stability. The IQM measurement linearity was evaluated in the 2–2000 MU range. IQM signal dose‐rate dependence was evaluated in the 15–405 MU/minute range, delivering 50 MU. The IQM signal was normalized using a Farmer type ion chamber (PTW, Freiburg, Germany) for the reference measurement. All ion chamber measurements were repeated at least in triplicate to evaluate standard deviation.

### 2.B Effect of the monitoring system on the treatment beam

The attenuating effect of the monitoring system on photon beams was measured by comparing the charge produced in a Farmer type ion chamber on the central axis of a 100 MU, 10 cm × 10 cm photon beam at 10 cm depth, with and without the device in place. Beam characteristics, especially PDD, can be affected by contaminating electrons. The production and path of contaminating electrons can be effected by the presence or absence of material and electric fields in the photon beam path. The effect of the monitoring system on the photon beam percent depth dose (%DD) and beam profiles was also characterized. Additionally, the measurements were repeated with the device in place but powered off, eliminating the presence of the ion chamber electric field, to evaluate the scenario of the device losing power during patient treatment. Percent depth doses and beam profiles were measured for a 30 cm × 30 cm field at 10 cm depth, with a CC13 ion chamber and the Blue Phantom water phantom (IBA, Bartlett, TN, USA). To evaluate the effect of the IQM on smaller IMRT‐sized photon beams, beam profiles were measured for a 1 × 1 cm^2^ field at 10 cm depth for each photon energy. These profiles were measured with an EDGE diode (SunNuclear, Melbourne, FL, USA). The effect of the monitoring system was characterized for 6 MV, 10 MV, and 15 MV photon beams.

### 2.C Photon beam error detection

The useful application of the IQM for online photon beam quality assurance requires that it must be able to detect clinically relevant errors in photon beam delivery. By modifying a simple photon field, the magnitude of signal produced in the ion chamber can be changed. In this work, these modifications are used to simulate treatment delivery errors. A 10 cm × 10 cm, representing a moderately sized normal field, and a 1 cm × 1 cm, representing a small‐sized field, with 6 MV photon beam were used as a baseline “correct” measurement. The magnitude of the modifications initially tested was based on the acceptable annual tolerances for photon output and MLC position reported in AAPM Task Group Report 142.^24^ The initial error simulations were 1% increase/decrease in MU, 1 mm single MLC leaf shift in/out of the field, 1 mm field shift in the MLC motion axis, 1 mm field shift perpendicular to MLC motion, and incorrect energy (10 MV and 15 MV). Each error simulation beam was measured in triplicate and the percent difference from the baseline measurement was reported. In cases where the mean IQM signal did not change more than twice the standard deviation of the baseline stability (1%), the magnitude of the modification was incrementally increased until the change in IQM signal was at least twice the standard deviation and the magnitude of detectable modification was reported.

### 2.D IMRT and VMAT reproducibility and simulated IMRT delivery error

As a baseline, the reproducibility of IMRT and VMAT measurements was characterized with triplicate measurements of a pharyngeal tonsil plan for IMRT and prostate plan for VMAT. Then, a well‐documented IMRT delivery error was selected to be simulated.^25^ In the selected case, a patient with tongue cancer was treated with static gantry IMRT. The photon beams were delivered with unmodulated open fields. An IMRT delivery error of a similar nature has been simulated by measuring the ion chamber signal of an anonymized nine‐field static MLC IMRT treatment for squamous cell carcinoma of the pharyngeal tonsil with field sizes 12–16 cm × 19 cm. The treatment was delivered with normal modulation and unmodulated and the ion chamber signal is compared.

## 3 Results and discussion

### 3.A Integrated quality monitoring system evaluation

The active area of the IQM covered the full area capable of being treated by the MLC and jaws, approximately 26 cm × 26 cm at the position of the detector (40 cm × 40 cm at isocenter). The IQM thermometer agreed to the calibrated thermometer to within 1.0 ± 0.7°C. The IQM barometer agreed to the mercury barometer to within 2.3 ± 0.4 mmHg. The IQM inclinometer gantry angle measurement agreed with the spirit level at 0 and 180 degrees within 0.03 ± 0.01 degrees and 0.27 ± 0.03 at 90 and 270 degrees. The IQM inclinometer gantry angle measurement agreed to the digital level at other angles within 0.24 ± 0.21 degrees. For the collimator angle measurement, the IQM inclinometer agreed with the plum‐bob within 0.3 ± 0.2 degrees with the gantry at 90 degrees. The inclinometer does not read out collimator angle when the gantry angle is within ~5 degrees of 0 or 180 degrees. This is likely because when the gantry is in this orientation, changes in collimator angle do not change the IQM inclination.

**Figure 2:**
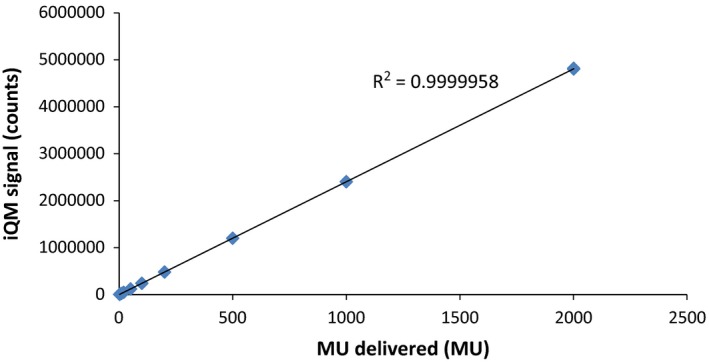
IQM signal shows linear dependence on the MU delivered for a 6 MV 10 cm × 10 cm photon beam.

**Figure 3:**
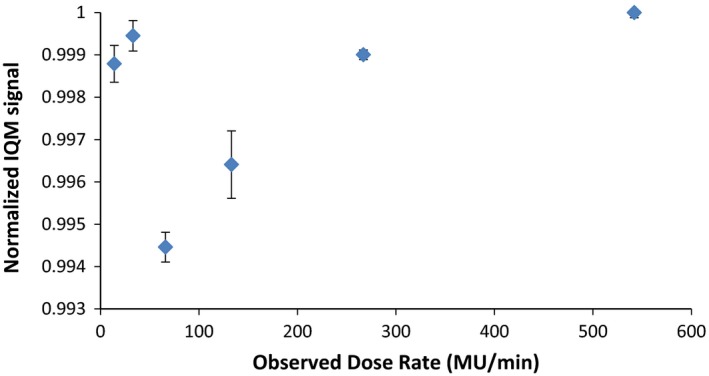
Normalized IQM signal measured from a 50 MU 10 cm × 10 cm 6 MV photon beam delivered at various dose rates. The measurements are normalized to the highest dose‐rate measurement with a Farmer chamber used as a reference measurement.

The 10 cm × 10 cm open beam measurements were reproduced with a standard deviation 0.14% on the same day of measurement. Measurements performed over 4 weeks varied with a standard deviation of 0.47%. IQM signal was linearly dependent on MU delivered (R^2^ = 1) as shown in Fig. 2. IQM signal initially showed a dose‐rate dependence of up to −4% at low dose rates, but after replacing one of the printed circuit boards with a different board incorporating a faster capacitor, this dose‐rate dependence was resolved, as shown in Fig. 3.

### 3.B Effect of the monitoring system on the treatment beam

**Figure 4:**
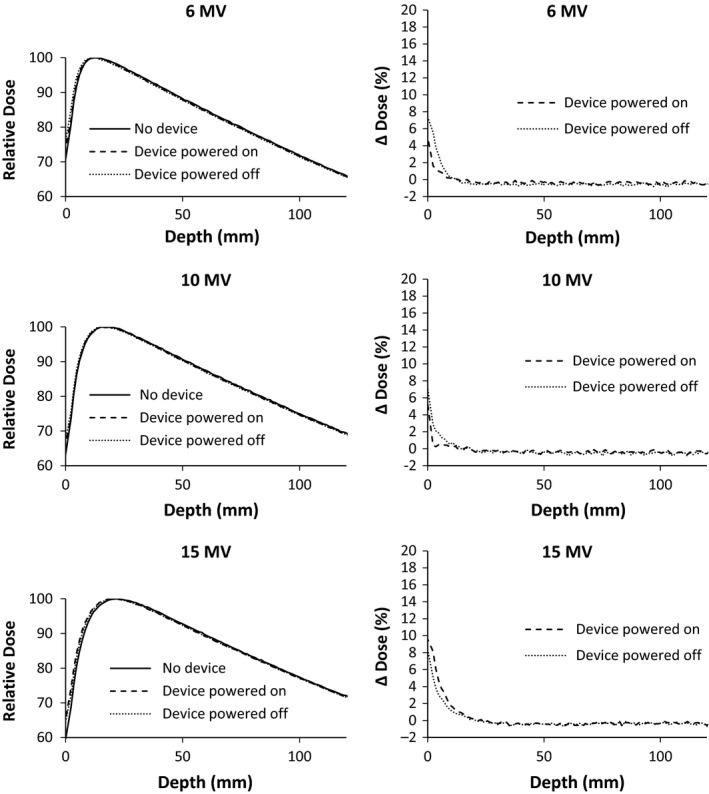
%DD of 10 cm × 10 cm photon beams of nominal energy 6 MV (top), 10 MV (middle), and 15 MV (bottom). %DDs were measured with and without the quality monitoring system in place and with it powered on and powered off. ∆ Dose (%) represents the percent difference from the %DD of the beam without the device in place.

**Figure 5:**
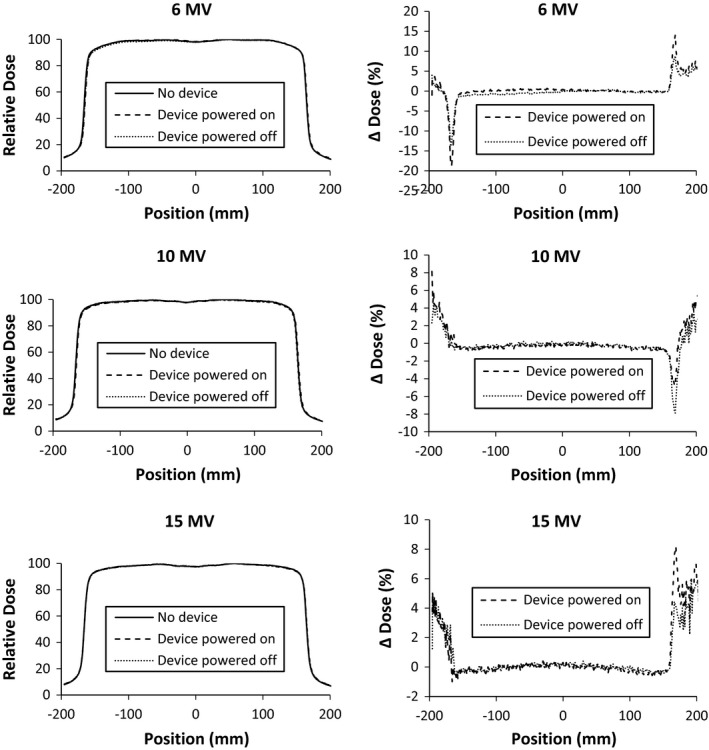
Beam profiles at 10 cm depth on the MLC motion axis of 30 cm × 30 cm photon beams of nominal energy 6 MV (top), 10 MV (middle), and 15 MV (bottom). Profiles were measured with and without the quality monitoring system in place and with it powered on and powered off. ∆ Dose (%) represents the percent difference from the profile of the beam without the device in place.

**Figure 6:**
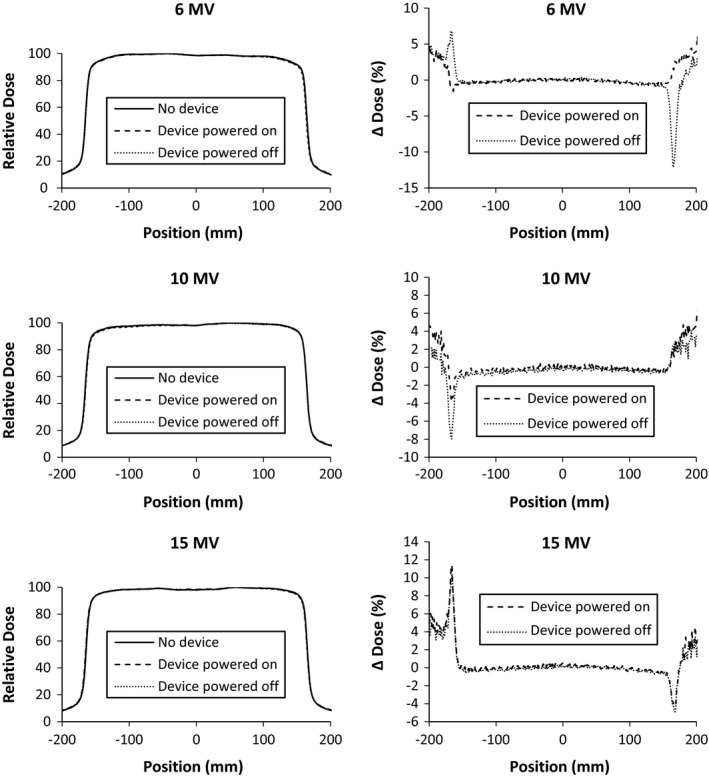
Beam profiles at 10 cm depth perpendicular to the MLC motion axis of 30 cm × 30 cm photon beams of nominal energy 6 MV (top), 10 MV (middle), and 15 MV (bottom). Profiles were measured with and without the quality monitoring system in place and with it powered on and powered off. ∆ Dose (%) represents the percent difference from the profile of the beam without the device in place.

**Figure 7:**
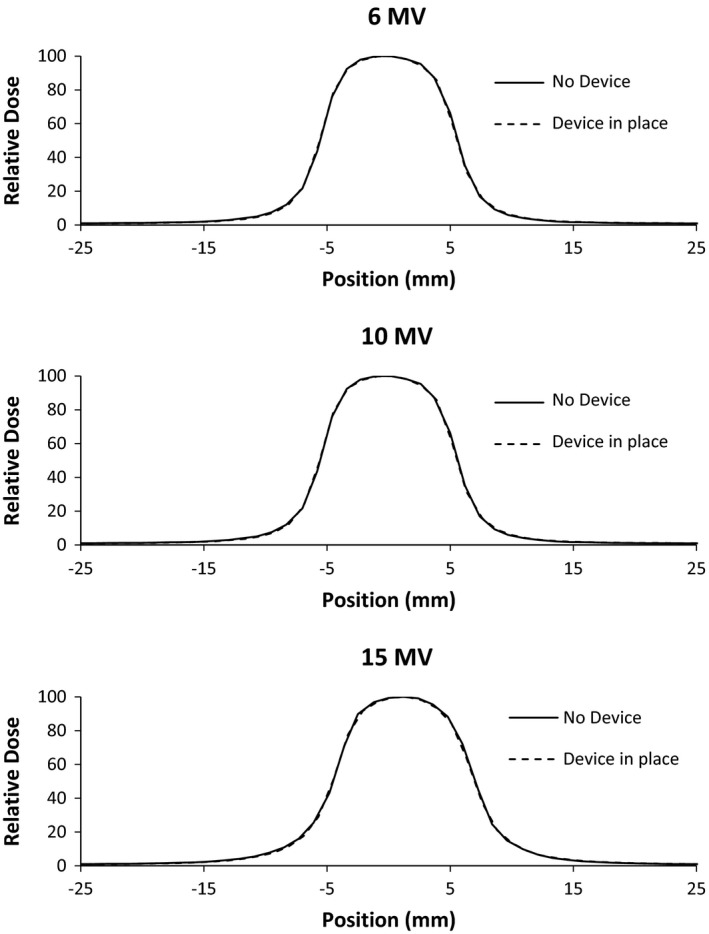
Inline beam profiles at 10 cm depth for 1 × 1 cm^2^ photon beams of nominal energy 6 MV (top), 10 MV (middle), and 15 MV (bottom). Profiles were measured with and without the quality monitoring system in place.

The presence of the large area detector attenuated 6 MV, 10 MV, and 15 MV photon beams 5.43 ± 0.02%, 4.60 ± 0.02%, and 4.21 ± 0.03%, respectively at depth of 10 cm in water. This attenuation was unchanged on the device being powered on or off. The effect on %DD in the buildup region is shown in Fig. 4. The presence of the IQM increased dose at shallow depths. At depths greater than 20 cm (not shown), all %DDs match within 0.5% for all energies. The change of the 10 × 10 cm^2^ beam profiles is shown in Figs. 5 and 6. The beam PDDs are modified by the presence of the IQM device, with increased % dose above depth of dose maximum. The symmetry and flatness of each beam profile is not changed due to the presence of the IQM. The nonpenumbra regions of the beams profiles agree generally within 1%. In the penumbra regions, the high‐dose gradient result in larger percent changes in dose (∆ dose %), but this represents submillimeter spatial change in dose. The inline profile of the 1 × 1 cm^2^ photon beam is similarly affected, as shown in Fig. 7. The crossline 1 × 1 cm^2^ profile is similarly unchanged. Each physicist commissioning this device would need to evaluate whether these changes in the beam characteristics and output can be accounted for with an attenuation factor or require the commissioning of the IQM attenuated photon beams for clinical use. This institution plans on accounting for the device with a tray factor, similar to the use of a graticule. The difference in %DD and profiles between the powered and unpowered IQM are shown in Fig. 4.

The dosimetric effects of the incorrect utilization of the device have not been investigated in this project. If the device was in place and not accounted for or accounted for while not in place, it would affect PTV dose in a way that is not characterized in this project.

### 3.C Photon beam error detection

The three baseline measurements of the normal (10 cm × 10 cm) field had similar reproducibility (0.15%) to the previously described reproducibility evaluation (0.14%), while the small (1 cm × 1 cm) field had 0.5% reproducibility. The percentage change measured from the baseline of each modification is listed in Tables 1 and 2. IQM signal changes equal or greater than 1% (more than twice the standard deviation of the stability of the measurement) were considered “detectable.” For both fields, increasing the number of MUs by 1%, decreasing the MUs by 1%, and increasing the nominal beam energy to 15 MV were detectable modifications. Moving a single MLC leaf 1 mm into and out of the field, and moving the field 1 mm in the MLC motion axis were not detectable modifications for either field. By increasing the magnitude of the modifications incrementally, we detected a 1.3 cm single leaf shift into the field, a 2.5 cm single leaf shift out of the field, and a 3 mm field shift in the MLC motion axis for the 10 cm × 10 cm field. The same process detected a 1.5 mm single leaf shift into and out of the field and a 4 mm field shift in the MLC motion axis for the 1 cm × 1 cm field.

**Table 1:** The percentage change of IQM signal when the baseline 6 MV photon beam, 10 × 10 cm^2^ field and 100 MU, is changed with the listed modifications. For modifications that result in less than a 1% signal change, the magnitude of modification to give 1% signal change is recorded

Modification	% signal change	Magnitude of modification for 1% change
1% decreased MU	−0.99 ± 0.01%	‐
1% increased MU	1.00 ± 0.03%	‐
1 mm single MLC leaf into field	−0.05 ± 0.01%	13 mm
1 mm single MLC leaf out of field	0.01 ± 0.01%	25 mm
1 mm field shift in MLC motion axis	0.42 ± 0.06%	3 mm
1 mm field shift in MLC nonmotion axis	0.20 ± 0.13%	Not sensitive
Incorrect energy (10 MV)	0.8 ± 0.02%	‐
Incorrect energy (15 MV)	2.85 ± 0.01%	‐

**Table 2:** The percentage change of IQM signal when the baseline 6 MV photon beam, 1 × 1 cm^2^ field and 100 MU, is changed with the listed modifications. For modifications that result in less than a 1% signal change, the magnitude of modification to give 1% signal change is recorded

Modification	% signal change	Magnitude of modification for 1% change
1% decreased MU	−1.1 ± 0.4%	‐
1% increased MU	1.02 ± 0.3%	‐
1 mm single MLC leaf into field	−0.7 ± 0.2%	1.5 mm
1 mm single MLC leaf out of field	0.5 ± 0.3%	1.5 mm
1 mm field shift in MLC motion axis	0.1 ± 0.3%	4 mm
1 mm field shift in MLC nonmotion axis	0.6 ± 0.4%	Not sensitive
Incorrect energy (10 MV)	8.5 ± 0.3%	‐
Incorrect energy (15 MV)	15.1 ± 0.3%	‐

The IQM is less sensitive to single MLC leaf changes in the 10 cm × 10 cm field than for the 1 cm × 1 cm field. The detected shifts for both fields represent ~0.65–1.25% changes in the irradiated area of the ion chamber to achieve a 1% change in ion chamber signal. This indicates that the sensitivity of the device to single MLC leaf changes increases as the field or segment size decreases. This scaling of sensitivity to field size has indications on the utility of the large area ion chamber's detection of MLC leaf position errors, especially compared to finite detector array technologies. Specifically, the IQM offers greater sensitivity for smaller radiation fields, such as in IMRT, but less precise single MLC leaf position accuracy in larger radiation fields.

Increasing the nominal beam energy to 10 MV resulted in a 0.8 ± 0.02% increase for the normal‐sized field, which falls below the 1% threshold (twice the stability standard deviation), but the nominal beam energy could not be incrementally increased to find a 1% change. Shifting the beam perpendicular to the MLC motion axis did not change the IQM signal in a position dependent fashion. This is a logical observation, as the ion chamber has no thickness gradient on this axis.

### 3.D Simulated IMRT delivery error

Repeated measurements of the pharyngeal tonsil IMRT plan resulted in a 0.15% standard in the checksum of each beam. Similarly, the repeated measurement of the prostate VMAT plan resulted in 0.16% standard deviation of the checksum for each arc. When the simulated IMRT error was delivered, the ion chamber signal of each segment increased by a factor of 11–238 times the baseline measurement, as shown in Table 3.

**Table 3:** The IQM signal of each segment for a representative beam of a pharyngeal tonsil IMRT plan. The plan was delivered correctly with the planed modulation and with a simulated error, where each segment of the plan was delivered without modulation and the MLC leaves open

Segment #	1	2	3	4	5	6	7	8	9
Correct delivery signal (counts)	45,836	23,544	26,631	555	2764	593	4305	11,122	10,538
Simulated error signal (counts)	490,104	286,685	585,695	158,296	421,544	141,033	78,807	394,401	276,328
% Difference	969%	1118%	2099%	28439%	15149%	23666%	1731%	3446%	2522%

## 4 Conclusion

Our investigation has demonstrated that the IQM is stable for online delivery quality assurance measurements. This device has been validated for reproducible measurements of 6 MV, 10 MV, and 15 MV photon beams. The IQM's signal linearity and dose‐rate dependence has been characterized and the dose‐rate dependence has been addressed. The IQM can detect deviations in MLC leaf position, and beam output (MU), and most photon beam energies from baseline measurements. The ion chamber signal has been evaluated to be dependent on photon beam output and MLC position modifications described in AAPM Task Group Report 142. Furthermore, it is an especially good candidate for monitoring small fields, because the device response is a checksum that does not depend on finite detectors which may not be small enough to detect fine MLC leaf position changes.

Future work will evaluate the reproducibility of checksum measurements for 3D conventional, IMRT, and VMAT plans, with a range of patient target volume sizes that covers the range of clinically relevant photon treatments.

## Acknowledgment

We acknowledge iRT Systems GmbH for use of the IQM device, software, and assistance. The authors also further acknowledge the support of Richard Valicenti and the University of California, Davis Department of Radiation Oncology, where this work was performed.

## Conflict of interest

The authors declare no conflict of interest.
